# Supplementary material for: Qubit vitrification and entanglement criticality on a quantum simulator
Source: Nat Commun. 2022 Dec 1;13:7395. doi: 10.1038/s41467-022-34982-3 (PMC9715663; doi:10.1038/s41467-022-34982-3)
Supplement: Supplementary file 1 — Supplementary Information [file 41467_2022_34982_MOESM1_ESM.pdf]

Supplementary information: Qubit vitrification and entanglement criticality on a quantum simulator

Jeremy Côté[1] and Stefanos Kourtis[1,*]

[1]Institut quantique & Département de physique, Université de Sherbrooke, Sherbrooke, Québec J1K 2R1, Canada

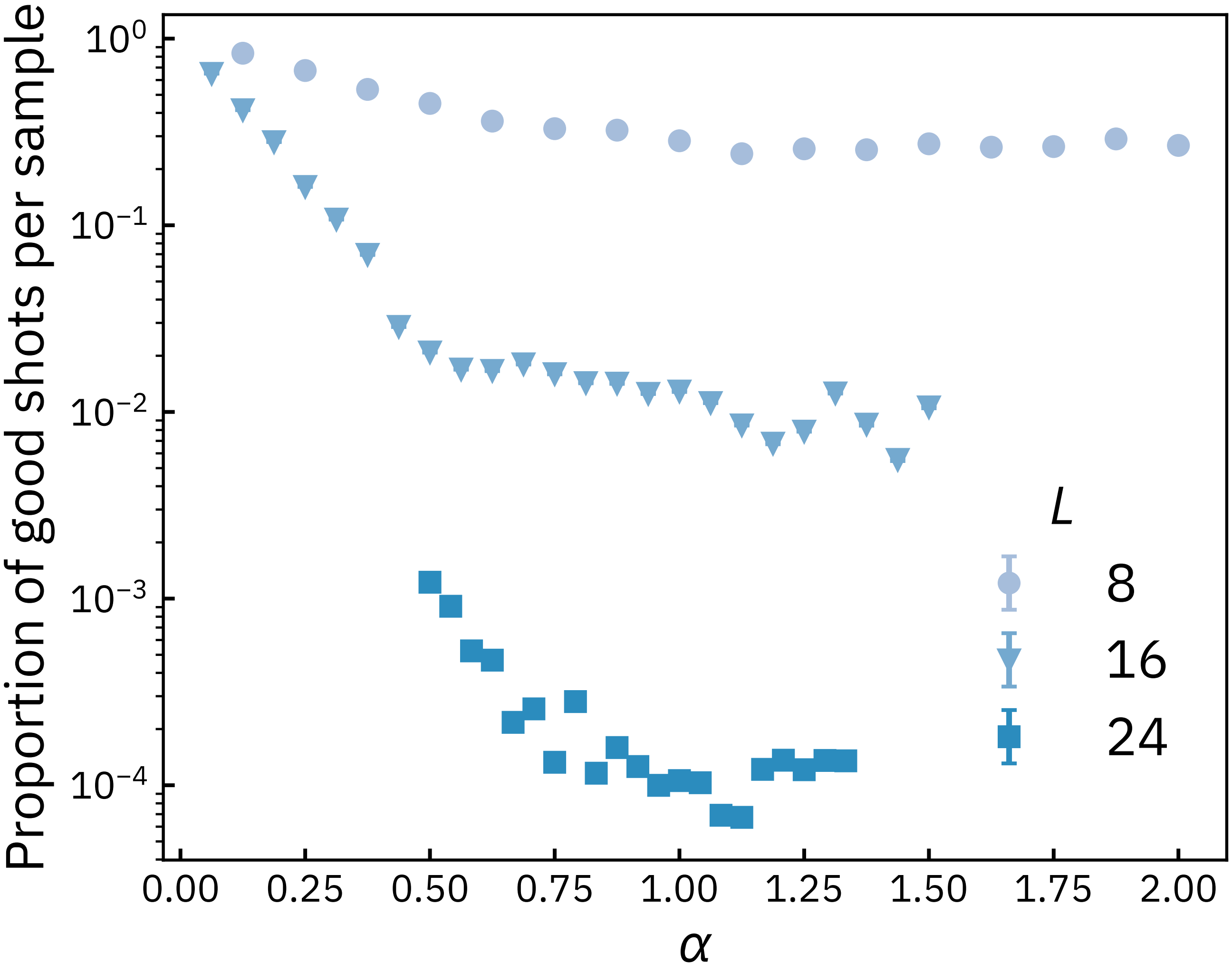

Supplementary Fig. 1: The proportion of shots which satisfy $B_{M'}\mathbf{x} = \mathbf{y}_{\mathrm{out},M'}$ for $|M| = L\alpha$ from our experiments on the IBM Q processors. Note the logarithmic vertical scale. We average over 900 samples for each data point, except for $L = 24$, where we average over 50 samples. Error bars indicate the standard error of the mean and are mostly smaller than the markers. We study system sizes $L = 8$ (light blue circles), $L = 16$ (medium blue triangles), and $L = 24$ (dark blue squares).

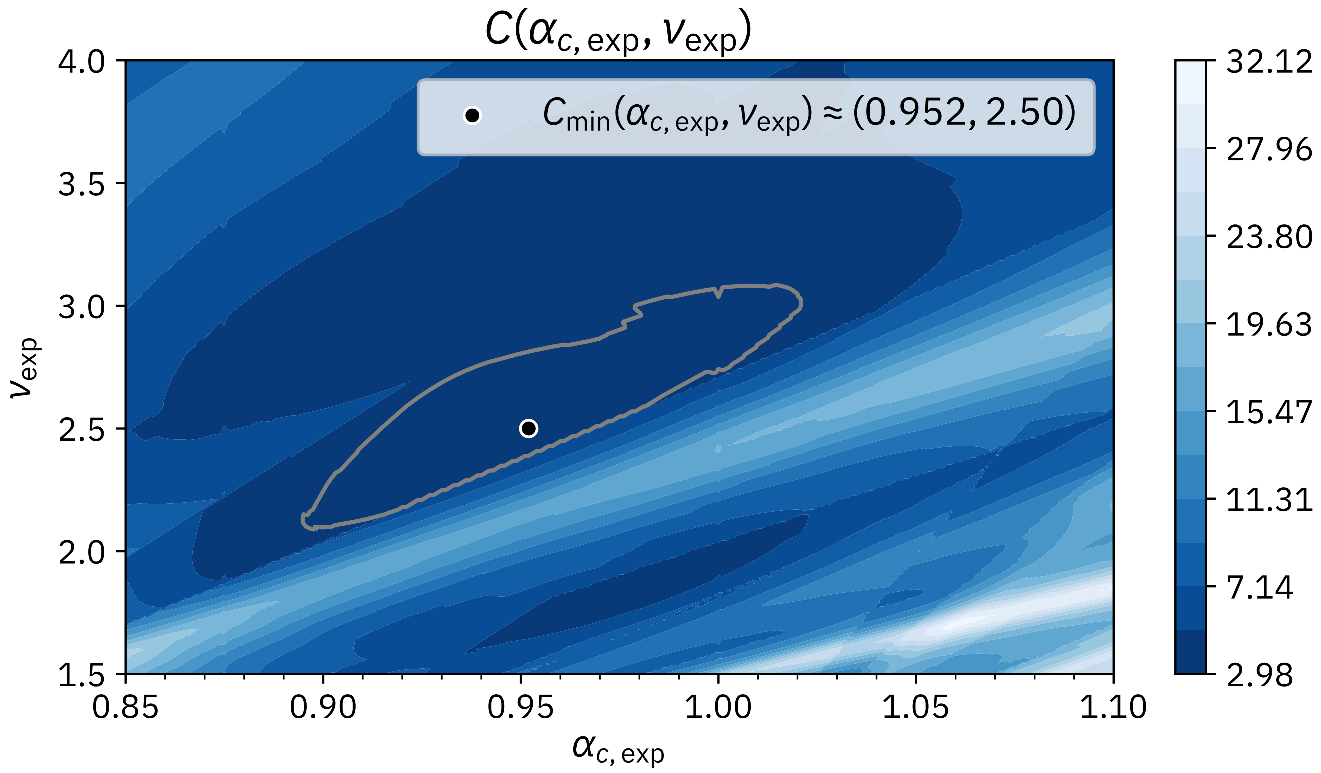

Supplementary Fig. 2: The cost function landscape over a grid of values of $\alpha_{c,\mathrm{exp}}$ and ν_{exp}. The black dot indicates the minimum of the cost function, located at the optimal $\alpha_{c,\mathrm{exp}}$ and ν_{exp} in the legend (to the resolution of the grid). The grey contour marks the region of uncertainty, given by $(1+r)\,C_{\mathrm{min}}(\alpha_{c,\mathrm{exp}}, \nu_{\mathrm{exp}})$, with $r = 0.25$. To compute $C(\alpha_{c,\mathrm{exp}}, \nu_{\mathrm{exp}})$, we used the range $\alpha_c \pm 0.5$ for the data in Equation (17).
